# Cognition and Alertness in Medical Students: Effects of a Single Night of Partial Sleep Deprivation

**DOI:** 10.1177/0972753120965083

**Published:** 2020-11-09

**Authors:** Priyadarshini Mishra, Madhuri Panigrahi, D. Ankit

**Affiliations:** 1 Department of Physiology, AIIMS, Bhubaneswar, Odisha, India; 2 AIIMS, Bhubaneswar, Odisha, India

**Keywords:** Sleep deprivation, cognition, reaction time

## Abstract

**Background:**

Partial sleep deprivation is common among young adults today. Though multiple studies have stressed on the benefits of having a good sleep, medical students often compromise their sleep due to academic targets and stress. This can lead to changes in attention and cognition. The effects of acute partial sleep deprivation of a single night have been studied less and studies in the past in Indian context have shown controversial results that reaction time is decreased following acute partial sleep deprivation.

**Purpose:**

The purpose of the study was to evaluate the effects of a single night of partial sleep deprivation on the cognitive status and alertness of medical students in the Indian context and to find out the change in auditory event-related potential (AERP) and psychomotor vigilance of medical students following a single night of partial sleep deprivation.

**Methods:**

The study was a before–after experimental trial conducted among 20 medical student volunteers of a tertiary care hospital of eastern India. Baseline psychomotor vigilance task measured by unprepared serial reaction time, and AERP measured by P300, were assessed at baseline (after normal sleep) and after four hours of sleep deprivation (intervention).

**Results:**

It was seen that median RT had increased from 320.4 ms to 337.6 ms after acute partial sleep deprivation (*P* < .001). P300 and lapses (*P* < .05) were also found to increase significantly (*P* < .05), while there was significant decrease in correctness (*P* < .01).

**Conclusion:**

The study concluded that cognition is affected, including alertness and latency, following partial sleep deprivation even for a single night and contradicted earlier results of Indian studies stating variable effect on psychomotor vigilance.

## Introduction

Sleep is a fundamental body requirement for a healthy life. Still, millions of people across the world are sleep-deprived.^1^ Sleep deprivation has been observed to have a detrimental effect on the physical and mental health and abilities of an individual,^2–4^ and it decreases attention span and performance.^5^ This finally involves various social, financial, and health-related costs.^6^ Partial sleep deprivation (PSD) or sleep restriction is a common condition that affects more than one-third of normal adults due to various factors including professional demands, social and domestic responsibilities, and sleep disorders.^2,7^ Effects of total sleep deprivation and a more common form of sleep deprivation, that is, acute PSD, have been extensively studied and demonstrated via various cognitive tests to produce decline in cognitive function.^7,8^ Medical students, comprising one of the most intelligent sections of the society, often compromise their sleep to perform well in academics. Findings of Pergher et al showed higher P300 amplitude and smaller latencies for subjects with a higher educational level.^9^ A higher cognitive reserve may compensate for neurocognitive deficits.^10^ Much less is known about the effect of PSD on the cognitive status and behavioral functions of this group. Moreover, studies in the past in the Indian context have shown controversial results that reaction time (RT) is decreased following acute PSD.^11^ Hence, this study was conducted with an aim to evaluate the effects of PSD on the cognitive status and alertness of medical students in the Indian context, and validate the earlier results.

## Methods

The study was conducted in the Department of Physiology, of a tertiary care center of eastern India, during May to July 2018, after permission from Institute Ethics Committee. It was a form of experimental study design (before and after study), where subjects served as their own controls. Intervention that was given was a single night of PSD. Participants were allowed to sleep for only 4 h (2–6 am). They were tested twice, once following a night of normal sleep (baseline) and again following a night of PSD (intervention). Considering unprepared serial RT as the primary variable for reference that determines psychomotor of an individual, and based on the results of a study conducted in India^9^ where the mean RT during baseline was 200.59 ± 34.59 ms and after intervention was 155.59 ± 36.24 ms, an effect size as drawn from this with correlation of 0.5 (being before and after) as 1.27 using G*Power 3.1 software, and sample size was calculated as 11 at 5% level of significance with statistical power of 95%. An additional sample of 20% was taken into account during recruitment considering the chances of drop-out mid-way during the study, and thus fixed as 14.

All students in each year of Bachelor of Medicine and Bachelor of Surgery (MBBS) were informed about details of the study through a common e-mail and invited for participation. Those who gave consent were given a chance to participate in the study. A brief clinical history was taken and examination performed for all students showing their willingness to participate in the study. Students with history of medical, neurological, hearing, or sleep related disorder, were excluded from the study. The remaining volunteers were given a sleep diary to be maintained for 2 weeks and their self-ratings of quality of sleep were also assessed using the Pittsburgh Sleep Quality Index (PSQI). Final selection of subjects was done on the basis of average PSQI scores (scores from 0 to 5 indicated good quality of sleep and from 6 to 21 indicated poor quality of sleep) and sleep diary. Volunteers having poor quality of sleep at night and having less than 7 hours of sleep at night, as evidenced from sleep diary and PSQI, were excluded from the study.

**Figure 1.**
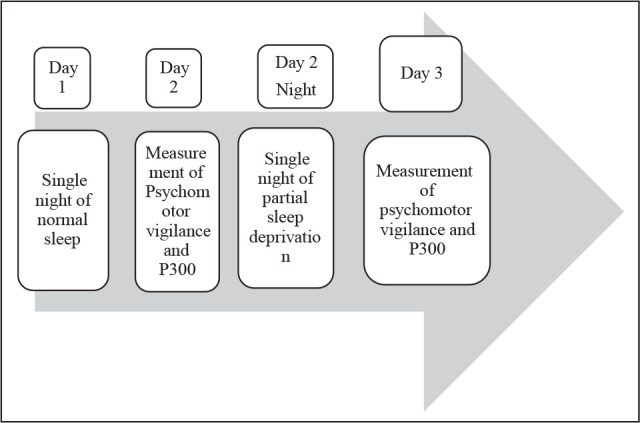
Study Design: Each Participant Underwent a 3-Day, Protocol

Before the study, participants were allowed to sleep for 3 nights in the laboratory to avoid bias due to unfamiliar surroundings during sleep. Before the study was started, participants were instructed to avoid stimulants like caffeine and also to avoid day time naps. Auditory event-related potential (ERP) (P300)^12,13^ and psychomotor vigilance task (PVT)^14^ were carried out on the subjects at 9:00 am on the day following a night of normal sleep, which was considered baseline. Each of the subjects then underwent PSD for a single night in the laboratory. Following this acute partial deprivation of night sleep, their P300 and PVT were estimated the next day at 9:00 am (Figure 1).

P300 was measured in quiet surroundings using Neuropack (Nihon Kodhen)^15^ as per the guidelines of the International Federation of Clinical Neurophysiologists. Event-related evoked potentials (P300) were recorded with Ag/Ag Cl electrodes from standard locations using 10–20 International system. the electrodes were placed at Fz, Cz, Pz (active electrodes at frontal, vertex, and parietal areas), FPz (ground electrode on the forehead), and A1, A2 (reference electrode behind the ear lobules). The recordings were obtained in response to standard auditory “odd-ball paradigm” where a frequent and a rare stimuli were given randomly. The skin electrode contact impedance was kept below 5Ω. The subjects were instructed to press a button on the response pad with the thumb of their dominant hand on hearing Auditory 1 (target, rare) among the frequently occurring stimuli delivered by headphones. During the recording session, subjects were instructed to fix his/her eyes on a particular spot on the wall in front in order to avoid electro-oculographic artifacts due to eye movements. Signals were averaged for 20 trials. The P300 wave was identified as the most robust positive wave between 200 and 400 ms after stimulus recognition. The peak latency and amplitude (base to peak) of the waveform was recorded and saved in the computer. Later these were entered in an MS Excel worksheet for data analysis.

PVT by the PEBL software^16^ version 0.14 (Shane Mueller August 2010) was utilized for PVT. The subject was seated comfortably asked to respond as soon as possible to red circle appearing in the center of the screen by pressing “Space Bar” on computer keypad. The red circle usually keeps on coming in the interval of 2–10 s. The unprepared serial RT was calculated in milliseconds. The total task duration was 10 min. Scores for PVT were noted under the following heads: average RT, number of lapses or errors of omission (RT > 500 ms), number of sleep responses (RT > 30 s), and number of too fast responses/errors of commission.

Data were entered onto a worksheet of MS Excel 2016, and imported to Stata version 12.1 SE. Data were presented either as categorical or continuous variables. Appropriate statistical tests were applied for analysis of the data. A *P* value of .05 was set as statistically significant. P300 amplitude and latency and auditory PVT data were compared using matched pair nonparametric tests (two-tailed sign test). Ethical clearance was obtained from the Institute Ethics Committee prior to the study. 

## Results

A total of 28 students expressed their consent to participate in the study and approached the investigators in response to the common e-mail sent. However, after maintaining the daily diary, it was seen that six students did not have adequate sleep that was considered for inclusion (>7 h). Two more students were excluded from the study because of poor quality of sleep (PSQI scores >6). Rest of the students were found to have a good quality of sleep ranging from 0 to 5 and were thus included in the study. Thus, a final total of 20 students were included in the experiment and were considered for analysis (*n* = 20). Median age of the study population was found to be 21.5 years (IQR: 21, 23 years). The study participants were predominantly males (*n* = 19, 95%).

Considering a small sample size of 20, nonparametric tests were used for analysis. Two-tailed sign test was used to compare the values before and after PSD. It was seen that the median average RT had increased from 320.4 ms to 337.6 ms after acute PSD, which was found to be very highly significant (*P* < .001). Statistically significant increase in P300 and lapses (*P* < .05) with highly significant decrease in correctness (*P*<0.01) were also seen among the study participants (see Table 1).

**Table 1.** Effect of Partial Sleep Deprivation Among Study Participants (*n* = 20)

Sl No.	Component	Pre-test Value Median (IQR)	Post-test Value Median (IQR)	*P* Value
1.	P300(latency in ms)	303.5 (284.0, 321.0)	309.5 (297.5, 337.0)	.0414*
	P300 (amplitude in µV)	25.89 (16.64, 31.39)	21.78 (17.93,27.63)	.16
2.	RT	320.4 (315.0, 332.3)	337.6 (326.6, 354.6)	.0004***
	Too fast	1.0 (0.0, 2.0)	2.0 (1.0, 3.0)	.1185
	Lapse	1.5 (0.0, 2.5)	3.0 (2.0, 4.0)	.0127*
	Correct	118.0 (117.0, 119.5)	117 (115,117)	.0013**

**Note:** *Significant at .05 levels. ** Highly significant at .01 level. *** Very highly significant at .001 levels. P300 = auditory event-related potential; RT = average reaction time, Too fast = number of too fast responses in PVT, Lapse = number of lapses (RT > 500 ms), Correct = number of correct responses.

**Figure 2.**
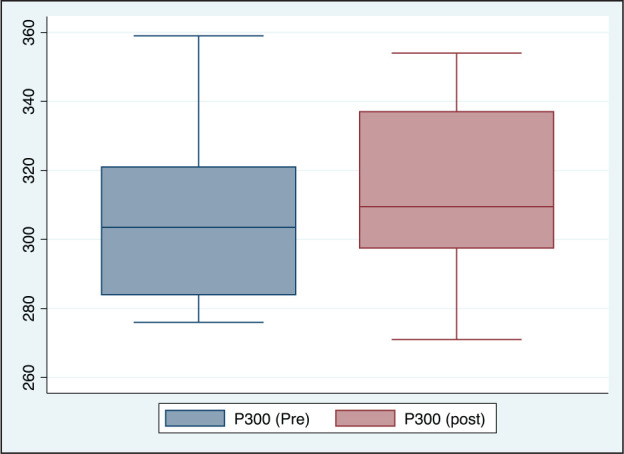
P300 (Latency) Before and After Acute Partial Sleep Deprivation Among Study Participants (*n* = 20)

**Figure 3.**
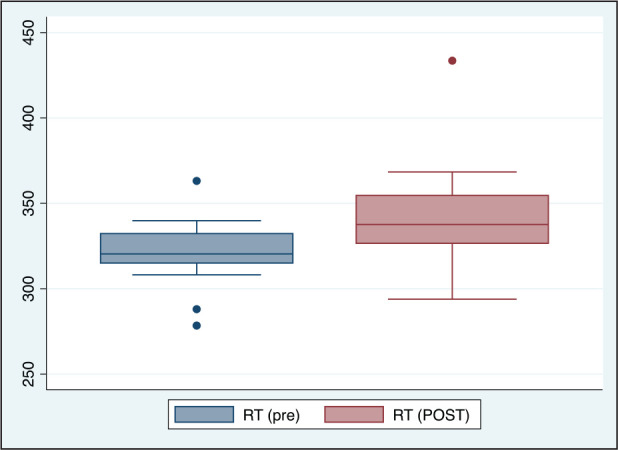
Comparison of Average Reaction Time (RT) Before and After Acute Partial Sleep Deprivation Among Study Participants (*n* = 20)

**Figure 4.**
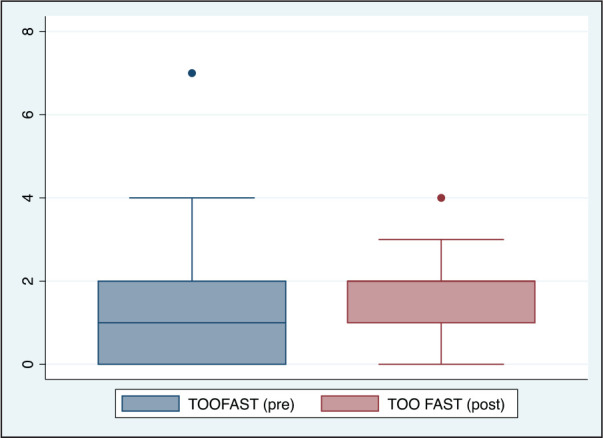
Comparison of Too Fast Responses (TOOFAST) Before and After Acute Partial Sleep Deprivation Among Study Participants (*n* = 20)

**Figure 5.**
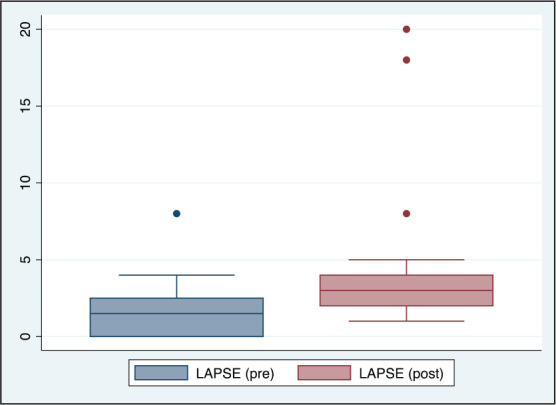
Comparison of Lapses (LAPSE) Before and After Acute Partial Sleep Deprivation Among Study Participants (*n* = 20)

**Figure 6.**
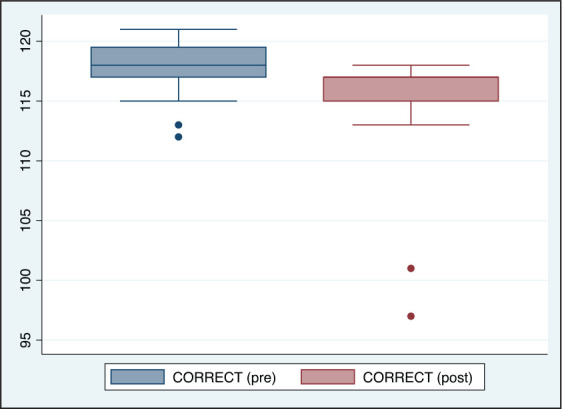
Comparison of Number of Correct Responses (CORRECT) Before and After Acute Partial Sleep Deprivation Among Study Participants (*n* = 20)

## Discussion

The difference in P300 was found to be marginally significant, with increase in PSD (Table1 and Figure 2). There were no significant differences between the amplitudes. The RT was found to be significantly high with increased number of lapses and decreased number of correct responses in case of PSD (Table 1, Figures 3, 4, 5 and 6).

Medical students have a rigorous curriculum and a highly competitive environment for academic excellence. In many situations, either during examinations or night duties, their sleep gets compromised. Acute PSD is more common than complete sleep deprivation in students. Educational background is a critical experience which develops over a period of time. The impact of formal education on cognitive ability has been explored in certain studies. ^10,17,18^ Higher education builds a cognitive reserve which can help compensate for conditions which affect neurocognition.^10^ Pergher et al. found higher P300 amplitudes and observed smaller P300 latencies for individuals that were more educated compared to less educated ones.^9^ Though the cognitive reserve is considered to be higher in students with formal education, our study revealed that PSD affects executive function and RTs in medical students.

The brain is affected by sleep or its deprivation. Synaptic plasticity and strength require sleep, as a consequence of which cognitive abilities such as learning and memory (especially long term^13^) are impaired following its deprivation.^15,19,17^ PSD, acutely and on a chronic basis, have been shown to have effects on cognition in many animal and human studies.^4,7,16,20–23^ Short periods of sleep restriction, say even an hour, after cognitive learning, can impair formation of memory.^24^

In a study conducted among medical students elsewhere in northern India,^11^ the P300 latency and amplitude were found to decrease significantly as compared to the test values at baseline. RT also showed a significant decrease in the test condition as compared to the baseline values. The study demonstrated that PSD produces variable effects on the cognitive status of medical students as reflected by the decrease in P300 amplitude and latency. Alertness of medical students seemed to show an improvement as reflected by the decrease in RT. This was contrasting to our study, where RT and latency of P300 was found to be significantly more in comparison to be baseline, signifying decrease in alertness and attention. The possible reasons may be inclusion of students with only good quality sleep in the current study and more controlled environment during the study.

Nap is often considered to be a powerful public health tool, and can reduce sleep-related accidents and improve performance.^25^ In a study trying to demonstrate the minimum effective duration of afternoon nap that could counteract acute PSD, it was seen that among the various durations of nap that can improve alertness and alertness following PSD, a 10 minute nap was the most effective among short nap durations that were considered in the study.^26^ In another study it was seen that nappers were able to tolerate frustration significantly longer than non-nappers. The latter reported feeling more impulsive after a 60 minute period.^27^ Day time napping was avoided in the current study. Thus, these medical students can be told about this 10-minute nap, which can be taken after the acute PSD during day time during lunch break, and even during the night time for increasing the attention span. However, chronic sleep deprivation needs to be avoided, and awareness created among the students, since it decreases long-term memory function as seen from various studies.

## Conclusion

The PVT and AERP were found to be significantly affected by acute PSD in the medical students. The RT and P300 were found to increase significantly as was the number of lapses (*P* < .05), while there was a highly significant decrease in correctness (*P* < .01) post intervention (sleep deprivation). This showed that a single night of PSD was also able to have effects on cognition levels among medical students.
